# The neural substrates responsible for food odor processing: an activation likelihood estimation meta-analysis

**DOI:** 10.3389/fnins.2023.1191617

**Published:** 2023-06-23

**Authors:** Nodoka Oka, Koichiro Iwai, Hiroyuki Sakai

**Affiliations:** Toyota Central R&D Labs., Inc., Nagakute, Japan

**Keywords:** olfactory, edibility, meta-analysis, human, chemosensory

## Abstract

In many species including humans, food odors appear to play a distinct role when compared with other odors. Despite their functional distinction, the neural substrates responsible for food odor processing remain unclear in humans. This study aimed to identify brain regions involved in food odor processing using activation likelihood estimation (ALE) meta-analysis. We selected olfactory neuroimaging studies conducted with sufficient methodological validity using pleasant odors. We then divided the studies into food and non-food odor conditions. Finally, we performed an ALE meta-analysis for each category and compared the ALE maps of the two categories to identify the neural substrates responsible for food odor processing after minimizing the confounding factor of odor pleasantness. The resultant ALE maps revealed that early olfactory areas are more extensively activated by food than non-food odors. Subsequent contrast analysis identified a cluster in the left putamen as the most likely neural substrate underlying food odor processing. In conclusion, food odor processing is characterized by the functional network involved in olfactory sensorimotor transformation for approaching behaviors to edible odors, such as active sniffing.

## 1. Introduction

Across many species including humans, who are considered highly visual animals, food odors appear to play a distinct role when compared with other odors. There is abundant evidence that food odors influence various eating behaviors such as appetite, choice, intake, and satiation (Boesveldt and de Graaf, 2017). Although this is not surprising since food odors constitute a strong sensory signal indicating the edibility of odor sources, such a close link between food odors and eating behaviors is involved in the social problem of obesity (Peng et al., 2019). Therefore, unveiling the mechanisms underlying food odor processing is a significant issue from the perspectives of both olfactory science and public health.

Despite their importance, the neural substrates responsible for food odor processing remain controversial in individual neuroimaging studies. Bragulat et al. (2010) examined brain activation in response to food and non-food odors and found that preferred food odors induced greater responses than non-food odors in extensive limbic and reward-related regions including the insula, anterior and posterior cingulate, ventral tegmental area, and ventral striatum. In a study conducted by Eiler et al. (2012) using a larger sample, food odors elicited greater activation in the medial prefrontal cortex, orbitofrontal cortex, and inferior insula than non-food odors. Frasnelli et al. (2015) further examined brain activation using food and pleasantness/intensity-matched flower odors but could not find any significant activation after multiple comparison corrections. Sorokowska et al. (2017) performed a region-of-interest analysis with small volume correction on functional magnetic resonance imaging (fMRI) data to identify food odor-related responses and revealed a significant involvement of the bilateral anterior cingulate cortex.

A meta-analysis is a powerful statistical tool for delineating the current state of knowledge from individual pieces of evidence. In the field of neuroimaging, activation likelihood estimation (ALE) is a well-established meta-analytical technique used to investigate the neural substrates of various sensory, motor, and cognitive functions (Eickhoff et al., 2012). Regarding olfactory processing, Zou et al. (2016) conducted an ALE meta-analysis and identified a core network for odor pleasantness that included the amygdala, middle frontal gyrus, and lateral orbitofrontal cortex. Furthermore, Torske et al. (2022) found in their ALE meta-analysis that food odors activated the bilateral putamen as well as the primary olfactory cortex. However, the confounding factors associated with food and non-food odors should be considered. In other words, food odors are likely to be more pleasant than non-food odors. To delineate the neural substrates responsible for food odor processing using an ALE meta-analysis, the activation of pleasant food odors should be contrasted with that of pleasant non-food odors (Frasnelli et al., 2015; Sorokowska et al., 2017).

In the current study, we thus aimed to identify the neural substrates responsible for food odor processing using an ALE meta-analysis. First, we selected olfactory neuroimaging studies conducted with sufficient methodological validity using pleasant odors. Second, we divided them into food and non-food conditions. Finally, we performed an ALE meta-analysis for each condition and compared the ALE maps of the two conditions to identify brain regions involved in food odor processing after minimizing the confounding of odor pleasantness.

## 2. Materials and methods

### 2.1. Study selection

**Figure 1:**
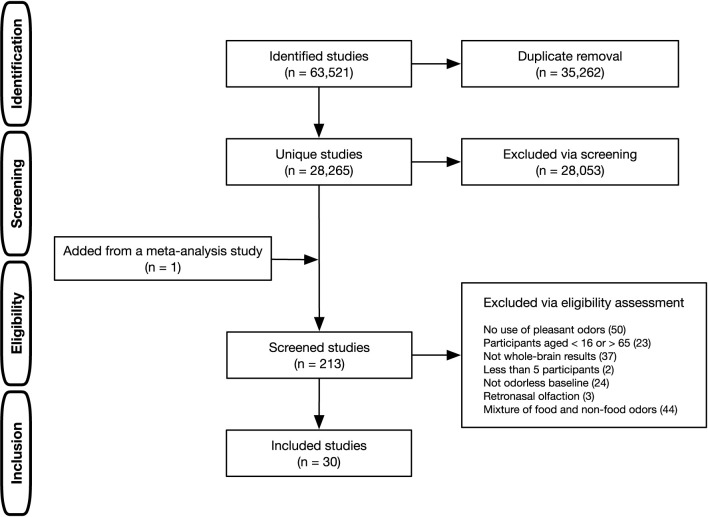
Literature selection process in the present ALE meta-analysis.

A multi-step procedure was adopted to identify literature relevant to the current research question (Figure 1). A literature search was conducted using PubMed, Web of Science, and ScienceDirect. The search terms were all combinations of two words (A and B), each selected from the following categories: (A) functional magnetic resonance imag*, fMRI, BOLD, regional cerebral blood flow, positron emission tomography, PET, and neuroimaging; (B) odor*, odour*, olfact*, and smell (the asterisk denotes a wildcard character). This comprehensive literature search identified 28,259 unique studies after removing duplicates. The first screening, based on titles and abstracts, excluded animal studies, meta-analyses, review articles, and non-olfactory studies (e.g., chemical treatment of odorants) and consequently narrowed down to 212 studies that were candidates for meta-analysis. To include studies not hit by our literature search, studies used in previous olfactory ALE meta-analyses (Seubert et al., 2013; Huerta et al., 2014; Zou et al., 2016; Torske et al., 2022) were checked, and one study by Ackerley et al. (2020) used in the most recent one (Torske et al., 2022) was added to the candidates (a total of 213 studies). Subsequently, further eligibility assessments were carried out. The inclusion criteria were only functional neuroimaging studies that used fMRI and PET, those that covered the whole brain, those that used the standard stereotactic coordinate space [i.e., Talairach or Montreal Neurological Institute (MNI)], and those with at least 5 or more healthy individuals aged 16 to 65. In addition to these general criteria, study-specific criteria were considered to identify distinct neural regions associated with odor edibility according to the guidelines proposed by Müller et al. (2018). Studies were included when orthonasal stimulation with pleasant odors was used, the mixture presentation of food and non-food odors was not examined, and isolated olfactory-evoked activation was identified by contrasting the odorless baseline. In this regard, multimodal studies were included only if the studies reported contrasts in which all non-olfactory effects were subtracted to isolate olfactory-evoked activation. These selection criteria were adopted because the current study aimed to separately identify olfactory activation to pleasant food and pleasant non-food odors. Finally, 30 studies met the inclusion criteria.

### 2.2. Contrast selection

From the 30 studies selected, 34 contrasts (232 foci) were extracted for our meta-analysis. They consisted of 18 contrasts (112 foci) for the food odor condition (Table 1 and Supplementary Table S1) and 16 contrasts (120 foci) for the non-food odor condition (Table 2 and Supplementary Table S2). Basically, only one contrast was selected from each study for each condition to avoid problematic dependence across contrasts (Turkeltaub et al., 2012). The only exception was a study by Boyle et al. (2007) from which two contrasts were included in the food condition. This was because those contrasts were respectively measured in two independent study populations (Boyle et al., 2007). Odors derived from vanilla and fruits were predominant in the food odor condition, whereas flower-derived odors such as rose and lavender were predominant in the non-food odor condition. Additional analyses on perceived pleasantness and edibility of these odors are provided in Supplementary material S1. The selected contrasts were inspected twice by one author (NO) and further checked by another author (HS) to ensure that the extraction was not subject to human error.

**Table 1:** Overview of the 18 contrasts (17 studies) classified as the food odor condition.

**Publication**	**Mode**	***N* (M/F)**	**Age**	**Foci**	**Odor**
**Alessandrini et al. (2014)**	**PET (^18^F-FDG)**	**11 (5/6)**	**45.7 ± 11**	**5**	**Vanillin**
Andersson et al. (2014)	fMRI	26 (0/26)	45 ± 13	12	Amyl acetate
Bengtsson et al. (2001)	PET (H_2_^15^O)	11 (11/0)	23–28	2	Vanillin
	PET (H_2_^15^O)	12 (0/12)	20–28	3	Vanillin
Eiler et al. (2012)	fMRI	18 (0/18)	26.1 ± 5.6	3	Pasta, roast beef
Han et al. (2018a)	fMRI	29	22.5 ± 2.6	6	Strawberries and cream, caramel, guava, orange
Han et al. (2018b)	fMRI	21 (8/13)	24.2	16	Pepper oil
Hillert et al. (2007)	PET (H_2_^15^O)	12 (0/12)	26 ± 3	8	Vanillin
Hoffmann-Hensel et al. (2017)	fMRI	20 (10/10)	25.2 ± 3.8	6	Orange, apple, chocolate, caramel
Lombion et al. (2009)	fMRI	15 (0/15)	20–23	11	Isoamyl acetate
Österbauer et al. (2005)	fMRI	9	27	6	Strawberry, lemon, spearmint, caramel
Reske et al. (2010)	fMRI	15 (0/15)	36.8 ± 7.7	3	Vanillin
Savic et al. (2002)	PET (H_2_^15^O)	12 (0/12)	20–28	2	Vanillin
Savic et al. (2009)	PET (H_2_^15^O)	12 (12/0)	21–36	3	Vanillin
Seo et al. (2013)	fMRI	25 (9/16)	23 ± 2	8	Bacon, strawberry
Small et al. (1997)	PET (H_2_^15^O)	10 (5/5)	22–41	6	Coffee, strawberry, grapefruit, soy source
Tubaldi et al. (2011)	fMRI	15 (7/8)	26	4	Orange, apple, strawberry, almond
Zou et al. (2018)	fMRI	25 (12/13)	19.8 ± 1.6	8	Pentyl acetate

The column “Mode” denotes neuroimaging methods. N, M, and F denotes the number of all participants, male participants, and female participants, respectively. The column ‘Foci' represents the number of reported foci.

**Table 2:** Overview of the 16 contrasts (16 studies) classified as the non-food odor condition.

**Publication**	**Mode**	***N* (M/F)**	**Age**	**Foci**	**Odor**
Ackerley et al. (2020)	fMRI	30 (15/15)	24 ± 3	11	Phenylethyl alcohol
Boyle et al. (2007)	fMRI	15 (15/0)	35.3	3	Phenylethyl alcohol
Eiler et al. (2012)	fMRI	18 (0/18)	26.1 ± 5.6	6	Douglas fir
Frasnelli et al. (2011)	PET (H_2_^15^O)	12 (0/12)	23.1	6	Polysantol
Han et al. (2018a)	fMRI	29	22.5 ± 2.6	2	Rose, olibanum, freesia, muguet
Hummel et al. (2013)	fMRI	19	INS: 23.2 ± 3.8, SEN: 26.3 ± 5.3	8	Ambroxan, mixture
Karunanayaka et al. (2014)	fMRI	10 (5/5)	24.7 ± 1.8	10	Lavender
Karunanayaka et al. (2015)	fMRI	18	27.0 ± 6.0	6	Lavender
Lombion et al. (2009)	fMRI	15 (0/15)	20–23	2	Phenylethyl alcohol
Masaoka et al. (2014)	fMRI	8 (4/4)	28.5 ± 8.3	4	Rose
Stankewitz et al. (2009)	fMRI	20	28	16	Rose
Treyer et al. (2006)	PET (H_2_^15^O)	9 (9/0)	20-31	6	Phenylethyl alcohol
Vedaei et al. (2017)	fMRI	15 (6/9)	30 ± 5	15	Eucalyptus
Wang et al. (2017)	fMRI	43 (26/17)	40.9 ± 15.0	8	Lavender
Wang et al. (2019)	fMRI	14 (9/5)	20.6 ± 2.1	2	Lavender
Wiesmann et al. (2006)	fMRI	22	27.0 ± 3.8	15	Phenylethyl alcohol

The column “Mode” denotes neuroimaging methods. N, M, and F denotes the number of all participants, male participants, and female participants, respectively. INS and SEN represent relatively insensitive and sensitive participants to a single odorant, respectively. The column “Foci” represents the number of reported foci.

Furthermore, demographic factors (age and biological sex) were examined as possible confounders. Specifically, the number of male participants, the number of female participants, and mean age were separately compared between the food and non-food conditions, using the Welch's *t*-test with a significance of *p* < 0.05. However, as shown in Tables 1, 2, not all studies provided information necessary for comparison. Studies lacking such necessary information were excluded in each comparison. In addition, when an age range for participants was provided, the median value of the range was used instead of the mean age. Results indicated no significant demographic biases between the two conditions [the number of male participants: *t*_(17.5)_ = 1.36, *p* = 0.19; the number of female participants: *t*_(24.3)_ = 0.90, *p* = 0.38; mean age: *t*_(30.4)_ = 0.42, *p* = 0.68].

### 2.3. Data analysis

The present meta-analysis was carried out using GingerALE 3.0.2 software implementing the ALE algorithm for neuroimaging results (https://brainmap.org/ale/). First, to conduct the meta-analysis in the MNI space, the coordinates reported in Talairach were converted to MNI using the Lancaster transform (Lancaster et al., 2007). Subsequently, each focus was modeled as a 3D Gaussian probability distribution centered at the given coordinates to account for spatial uncertainty. The width of the uncertainty function was determined based on empirical data of the between-subject and between-template variances. Importantly, the applied algorithm weights the between-subject variance by the number of examined subjects, accommodating the notion that larger sample sizes should provide more reliable approximations of activation areas and should therefore be modeled by smaller Gaussian distributions. The ALE maps for the food and non-food odor conditions were thresholded using a voxel-level threshold of uncorrected *p* < 0.001 for cluster-formation and regarded as significant at *p* < 0.05 family-wise error corrected for multiple comparisons with 1,000 permutations (Eickhoff et al., 2017). In addition, an ALE map was also created from the pooled data from both the food and non-food odor conditions using the identical statistical criteria. Finally, contrast analysis was conducted using the above three separate ALE maps (i.e., food, non-food, and pooled) to identify the specific processing nodes for the food and non-food odor conditions, respectively. The results were reported with a voxel-level threshold of *p* < 0.01 with 10,000 permutations.

Moreover, a leave-one-out jackknife sensitivity analysis (Lyles and Lin, 2010) was carried out to examine the robustness of findings in the current ALE meta-analysis. Specifically, the above ALE analysis was iterated by excluding one contrast at a time. Then, it was verified whether brain regions found in the original ALE analysis were reliably found across the iterations. In addition, one may be concerned that the inclusion of PET studies would bias the results due to modality-related differences, such as spatial resolution and image processing procedures (Li et al., 2022). To rule out this possibility, the ALE analysis was also performed using fMRI studies only.

## 3. Results

**Figure 2:**
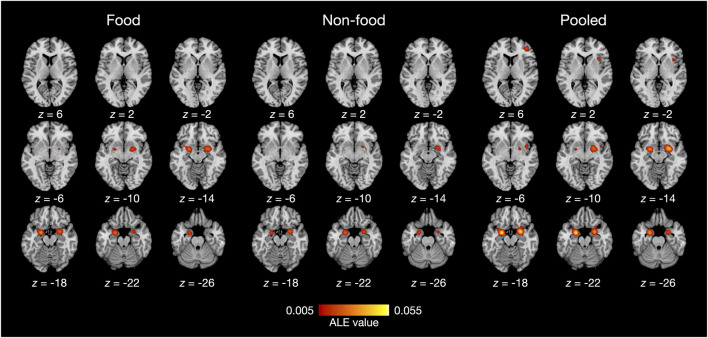
ALE meta-analysis results of olfactory neuroimaging studies with food odors and non-food odors. Statistical significance was set at family-wise error corrected *p* < 0.05 with a voxel-level threshold of uncorrected *p* < 0.001 for cluster-forming. The axial slices are displayed in neurological orientation (right on right).

The results of the ALE meta-analysis are summarized in Table 3 and Figure 2. A meta-analysis of 18 contrasts classified as the food odor condition delineated bilateral activation in early olfactory areas, including the piriform cortex, amygdala, and entorhinal cortex (BA28, BA34). Bilateral activation in early olfactory areas was also observed in a meta-analysis of 16 contrasts classified as the non-food odor condition. Additionally, a meta-analysis was conducted by combining both the food and non-food odor conditions (34 contrasts in total) to delineate the regions activated by pleasant odors. The results showed significant activation in the right insular (BA13) and prefrontal regions (BA10, BA46), as well as in the early olfactory structures in both hemispheres.

**Table 3:** All clusters resulting from the present ALE meta-analysis.

		**Peak locus**					
**Cluster**	**Volume (mm** ^3^ **)**	*x*	*y*	*z*	**ALE value**	**Anatomical label**	**JS**
**Food**							
1	3,616	−22	−2	−18	0.0324	Amygdala, BA34, BA28, Putamen, Lateral globus pallidus	18/18
2	3,072	26	0	−16	0.0261	Putamen, Amygdala, Lateral globus pallidus, BA34, Medial globus pallidus, BA28	18/18
**Non-food**							
1	2,120	26	2	−18	0.0243	BA34, Amygdala, Putamen, Lateral globus pallidus	16/16
2	1,488	−22	−2	−22	0.0250	Amygdala, BA34, BA28	16/16
**Pooled**							
1	5,016	26	2	−18	0.0476	Putamen, Amygdala, BA34, Lateral globus pallidus, Medial globus pallidus, BA28	34/34
2	4,104	−22	−2	−20	0.0534	Amygdala, BA34, BA28, Putamen, Lateral globus pallidus	34/34
3	816	36	16	2	0.0162	BA13	29/34
4	792	42	44	6	0.0212	BA10, BA46	30/34
**Food** > **non-food**							
1	40	−22	2	−10		Putamen	23/34

JS, jackknife sensitivity; BA, brodmann area.

**Figure 3:**
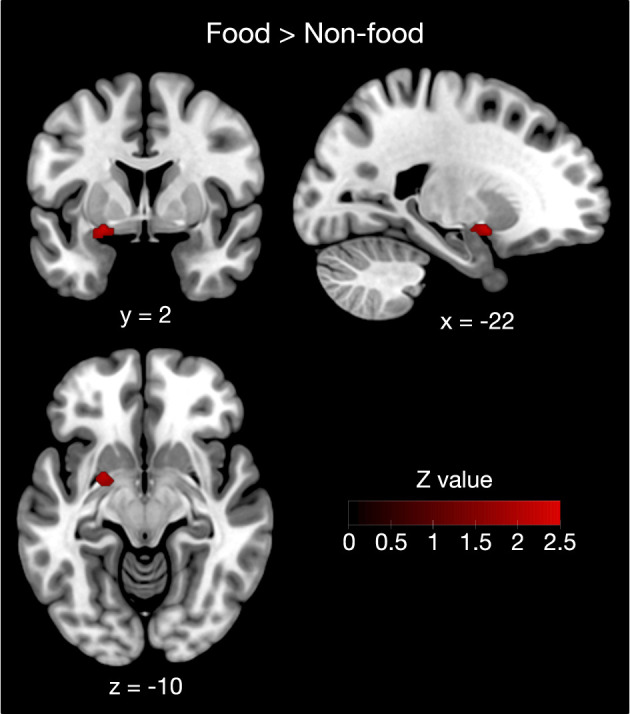
Contrast analysis between the food and non-food odor conditions. The left putamen was more likely activated in food rather than non-food odors. Results were reported as significant with a voxel-level threshold of *p* < 0.01, although this figure was depicted with a more liberal statistical criterion (*p* < 0.05) for visualization purpose.

Furthermore, contrast analysis revealed a significant cluster in the left putamen (Table 3 and Figure 3). This cluster entirely overlapped with the left hemisphere cluster found in the food odor condition (Table 3 and Figure 2) and is more likely to be activated in the food than the non-food condition. In contrast, no regions showed significant activation in the opposite contrast. Even using a more liberal statistical criterion (i.e., *p* < 0.05), these findings remained unchanged, except for expanding the left putamen cluster to include the lateral globus pallidus (Figure 3).

The jackknife sensitivity analysis (Table 3 and Supplementary Table S3) revealed that bilateral activation in early olfactory areas was robust in any combination of studies. The right insular and prefrontal regions were found in almost all study combinations. In contrast, the involvement of the left putamen was replicated in 23 out of 34 study combinations, indicating moderate robustness of the result. Moreover, the findings were substantially robust even when PET studies were excluded (Supplementary Table S4).

## 4. Discussion

Despite their functional importance, the neural substrates underlying odor edibility remain unclear. To address this question, we performed an ALE meta-analysis of the existing olfactory neuroimaging studies using food and non-food odors. Consequently, our ALE meta-analysis demonstrated that the early olfactory areas were more extensively activated by food than non-food odors. Our meta-analysis further identified a cluster in the left putamen as the most plausible neural substrate underlying food odor processing with moderate robustness. Moreover, an additional meta-analysis using the pooled data found that the insular and prefrontal cortices in the right hemisphere were activated by pleasant odors.

In all of our individual ALE meta-analyses, we observed robust bilateral activation in the limbic and basal ganglia regions, including the piriform cortex, amygdala, and parahippocampus. Because these regions constitute the early olfactory system receiving strong projections from the olfactory bulb (Haberly, 2001), it is not surprising that they showed high activation probabilities regardless of odor quality. In fact, activation in these regions has been repeatedly observed in response to plants (Han et al., 2018a), food (Howard and Gottfried, 2014; Bhutani et al., 2019), and perfume ingredients (Plailly et al., 2012), as well as in previous meta-analyses of the olfactory system in the brain (Seubert et al., 2013; Huerta et al., 2014; Zou et al., 2016; Torske et al., 2022).

The larger activation of early olfactory structures in the food rather than the non-food odor condition was a more important observation in the current study. Although a few studies have sought to identify the neural substrates responsible for food odor processing, none have reported a markedly greater involvement of these regions (Eiler et al., 2012; Frasnelli et al., 2015; Sorokowska et al., 2017). Instead, previous studies have reported the involvement of frontal regions, such as the medial prefrontal cortex, in food odor processing (Eiler et al., 2012; Sorokowska et al., 2017). However, they also demonstrated that differences in activation in response to food and non-food odors were rather subtle because no such regions survived whole-brain level correction for multiple comparisons.

The left putamen found in the contrast analysis was part of the enlarged activation in response to food odors compared with non-food odors. Although the putamen is known to play a crucial role in motor control (DeLong et al., 1984), putaminal activation has frequently been reported in olfactory neuroimaging studies. For example, Han et al. (2018b) showed that essential oil extracted from black pepper activates the left, but not the right, dorsal striatal structures including the putamen. In addition, it has been demonstrated that putaminal dopamine function is associated with olfactory perceptual sensitivity (Larsson et al., 2009). However, previous studies exploring the neural substrates of odor edibility have not shown that putamen activation is associated with odor edibility (Eiler et al., 2012; Frasnelli et al., 2015; Sorokowska et al., 2017). In a previous meta-analysis (Torske et al., 2022), a small cluster in the left putamen was likely activated by food rather than non-food odors. This seems consistent with our results, although contrasting food with non-food odors, as in a previous study (Torske et al., 2022), may have a confounding influence on pleasantness. Our data demonstrated that the left putamen is most likely the neural substrate of food odor processing by comparing pleasant food and pleasant non-food odors.

A recent neuroimaging study by Zhou et al. (2019) provided intriguing insights into the olfactory role of the putamen based on functional connectivity analysis of the piriform cortex. This study revealed that the frontal subregion of the piriform cortex constitutes a distinct functional network with motor planning areas, such as the caudate/putamen and the facial movement-related areas in the primary motor cortex. Thus, they proposed a novel hypothesis that this functional network comprising the putamen may play a specific role in guiding motor actions (e.g., sniffing) in response to food odors by transforming olfactory information to motor planning. This putaminal role in olfactory sensorimotor transformation is consistent with our finding that food odors likely activate extensive areas of early olfactory structures, including the putamen.

Nonetheless, our data does not indicate that the putamen is the only region involved in food odor processing. In fact, when using a somewhat liberal statistical criterion (i.e., *p* < 0.05), the significant cluster expanded to include not only the putamen but also the globus pallidus. The most accepted function of the globus pallidus is to control conscious and proprioceptive movements (Javed and Cascella, 2023). However, its primary role in the olfactory system is not fully understood. According to a previous meta-analysis (Zou et al., 2016), the globus pallidus is considered to be a part of the core olfactory hedonic processing network. In addition, evidence from an animal study demonstrated that a lesion of the globus pallidus causes decreased sniffing activity in an experimental chamber as well as decreased locomotor activity in an open field (Hauber et al., 1998). This suggests that the globus pallidus may have a role in the expression of olfactomotor behaviors, which is consist with the abovementioned putaminal role in olfactory sensorimotor transformation.

Although there is abundant evidence that the orbitofrontal cortex (OFC) is the core region of the olfactory system (Gottfried and Zald, 2005), the current ALE meta-analysis did not find any significant involvement of the OFC. However, this does not prove the irrelevance of the OFC in odor edibility and/or pleasant odor processing. Rather, even in the studies included in the current meta-analysis, OFC activation was repeatedly reported (Small et al., 1997; Österbauer et al., 2005; Treyer et al., 2006; Boyle et al., 2007; Frasnelli et al., 2011; Tubaldi et al., 2011; Masaoka et al., 2014; Hoffmann-Hensel et al., 2017; Vedaei et al., 2017). Accordingly, the absence of findings in the OFC may be due to the spatial uncertainty resulting from methodological difficulties in measuring OFC activity using fMRI (Zald and Pardo, 2000). Specifically, the field inhomogeneity around the OFC is known to damage the quality of fMRI signals due to, for example, signal dropout and geometric distortions (Ojemann et al., 1997), causing increased spatial uncertainty of olfactory OFC activation. Although imaging techniques have been developed to overcome this problem (Weiskopf et al., 2006, 2007), there is still no generally accepted acquisition solution. To properly evaluate OFC involvement in olfactory food processing, susceptibility issues during image acquisition are a major technical challenge to be solved.

This study has some limitations which need to be acknowledged. First, we carefully selected olfactory neuroimaging studies using only pleasant odors to avoid the confounding of odor pleasantness between food and non-food odors. Therefore, further attempts are required to validate the generality of our results using studies on unpleasant odors. However, it would be difficult to collect a sufficient number of such studies with food odors to perform ALE analysis. Second, it was extremely difficult to prove in our retrospective meta-analysis that pleasantness was comparable between food and non-food odors used in the selected studies. Therefore, we cannot completely rule out the possibility that biases regarding pleasantness (and also intensity) remained. Third, we performed ALE analysis using only conventional activation studies. This methodology is ineffective when odor edibility is encoded by activation patterns within certain regions. To overcome this issue, an ALE meta-analysis should be performed using studies with decoding approaches, such as multi-voxel pattern analysis (Norman et al., 2006).

## 5. Conclusion

The extensive activation of early olfactory structures, including the putamen, is associated with odor edibility. This suggests that odor edibility is characterized by the functional network involved in olfactory sensorimotor transformation for approaching behaviors toward edible odors, such as active sniffing. This is a fascinating hypothesis regarding the neural substrate of odor edibility, but requires further validation in future studies. In addition, our findings emphasize the need for olfactory neuroimaging studies with more sophisticated experimental and analytical methods to reveal the spatial encoding of odor quality.

## Data availability statement

The raw data supporting the conclusions of this article will be made available by the authors, without undue reservation.

## Author contributions

NO and HS participated in the design of the study, analyzed all the research data, and drafted the manuscript. KI reviewed the manuscript. All authors read and approved the final manuscript.
