# Supplementary material for: The neural substrates responsible for food odor processing: an activation likelihood estimation meta-analysis
Source: Front Neurosci. 2023 Jun 23;17:1191617. doi: 10.3389/fnins.2023.1191617 (PMC10326844; doi:10.3389/fnins.2023.1191617)
Supplement: Supplementary file 1 [file Data_Sheet_1.pdf]

Supplementary Material

S1. PERCEIVED PLEASANTNESS AND EDIBILITY OF ODORS USED IN THE SELECTED STUDIES

In the current activation likelihood estimation (ALE) meta-analysis, we selected studies using pleasant food and pleasant non-food odors to identify brain regions responsible for food odor processing. Here, we compared perceived pleasantness and edibility of odors in the food and non-food odor conditions using an available dataset published by Keller and Vosshall (2016). This dataset consists of olfactory perception scores of 480 structurally and perceptually diverse odorant molecules. We first extracted molecules used in the selected studies from the dataset. We were able to find all single odorants used except polysantol. Moreover, we also extracted major components of odors as follows: d-limonene for citrus odors (e.g. orange, lemon, grapefruit), benzaldehyde for almond odor, (-)-carvone for spearmint odor, 2-phenylethanol for rose odor, linalool and linalyl acetate for lavender odor, camphor for rosemary odor, 1,8-cineole for rosemary and eucalyptus odors, and alpha-pinene and beta-pinene for douglas fir odor. Then, we compared pleasantness and edibility scores of extracted molecules between the food and non-food odor conditions using the Welch's t-test with a significance of $p < 0.05$. Results revealed that pleasantness scores were comparable [$t(10.4) = 1.31$, $p = 0.218$] between the food (M = 66.3, SD = 13.1) and non-food odor conditions (M = 57.3, SD = 12.2). In contrast, edibility scores were significantly higher in the food (M = 14.0, SD = 6.36) compared with the non-food (M = 3.84, SD = 2.64) condition [$t(6.3) = 3.66$, $p = 0.010$]. These results suggest that our study selection worked as expected, although this analysis did not encompass all odorants used in the selected studies (e.g. pasta, roast beef, olibanum, freesia).

Table S1. Detailed information of studies in the food odor condition.

	Task	Inhalation	Statistics	Remarks
Alessandrini et al. (2014)	None	Passive	Uncorrected $p < 0.05$ at voxel level and corrected $p < 0.05$ at cluster level	Participants were fasted for at least 5 hours before FDG injection.
Andersson et al. (2014)	Odor rating	Passive	FWE-corrected $p < 0.05$ at voxel level with an extent threshold of 10	
Bengtsson et al. (2001)#1	None	Passive	Uncorrected $p < 0.01$ at voxel level and corrected $p < 0.05$ at cluster level	
Bengtsson et al. (2001)#2	None	Passive	Uncorrected $p < 0.01$ at voxel level and corrected $p < 0.05$ at cluster level	
Eiler et al. (2012)	Odor detection	Passive	Uncorrected $p < 0.001$ at voxel level and FWE-corrected $p < 0.005$ at cluster level	Both hungry and satiety participants were included.
Han et al. (2018a)	Visual detection	Passive	FDR-corrected $p < 0.05$ at voxel level	Participants were served a standard breakfast after a 12-hour fast and scanned 3 hours after breakfast.
Han et al. (2018b)	None	Passive	Uncorrected $p < 0.005$ at voxel level with an extent threshold of 10	
Hillert et al. (2007)	None	Passive	Corrected $p < 0.05$ at cluster level with a T-threshold of 0.01	
Hoffmann-Hensel et al. (2017)	Language memory	Passive	FWE-corrected $p < 0.05$ at voxel level	Participants were fasted (excluding water) for at least 1 hour before the experiment
Lombion et al. (2009)	None	Passive	Uncorrected $p < 0.001$ at voxel level	
Österbauer et al. (2005)	Odor detection	Passive	Corrected $p < 0.05$ at cluster level with a Z-threshold of 2.0	
Reske et al. (2010)	None	Passive	Uncorrected $p < 0.001$ with an extent threshold of 10	
Savic et al. (2002)	None	Passive	Corrected $p < 0.05$ at cluster level	
Savic et al. (2009)	None	Passive	Corrected $p < 0.05$ with a T-threshold of 0.001	
Seo et al. (2013)	None	Passive	Uncorrected $p < 0.005$ with an extent threshold of 3	
Small et al. (1997)	Odor detection	Passive	Uncorrected $p < 0.0002$ at voxel level	
Tubaldi et al. (2011)	Visual viewing	Passive	FDR-corrected $p < 0.015$ with an extent threshold of 15	
Zou et al. (2018)	Olfactory incentive delay	Active	Uncorrected $p < 0.001$ with an extent threshold of 10	

The column ‘Inhalation’ indicates instruction to participants regarding odor inhalation. Specifically, studies were labeled as ‘Active’ if participants were clearly instructed to sniff odor stimuli; in contrast, studies were labeled as ‘Passive’ if participants were not instructed to sniff or were instructed to maintain spontaneous breathing. Abbreviations: FWE, family-wise error; FDR, false discovery rate.

Table S2. Detailed information of studies in the non-food odor condition.

	Task	Inhalation	Statistics	Remarks
Ackerley et al. (2020)	None	Passive	Uncorrected $p < 0.001$ with an extent threshold of 20	
Boyle et al. (2007)	None	Passive	Uncorrected $p < 0.001$ with an extent threshold of 3	
Eiler et al. (2012)	Odor detection	Passive	Uncorrected $p < 0.001$ at voxel level and FWE-corrected $p < 0.005$ at cluster level	Both hungry and satiety participants were included.
Frasnelli et al. (2011)	None	Passive	Corrected $p < 0.05$ at cluster level with a T-threshold of 3.0	Participants refrained from eating, drinking, and smoking other than water for 1 hour before the experiment.
Han et al. (2018a)	Visual discrimination	Passive	FDR-corrected $p < 0.05$ at voxel level	Participants were served a standard breakfast after a 12-hour fast and scanned 3 hours after breakfast.
Hummel et al. (2013)	None	Passive	Uncorrected $p < 0.001$ at voxel level and FWE-corrected $p < 0.05$ at cluster level	
Karunanayaka et al. (2014)	None	Passive	FWE-corrected $p < 0.05$	
Karunanayaka et al. (2015)	Multi-modal detection	Passive	Uncorrected $p < 0.001$ at voxel level	
Lombion et al. (2009)	None	Passive	Uncorrected $p < 0.001$ at voxel level	
Masaoka et al. (2014)	None	Passive	FWE-corrected $p < 0.05$ at voxel level	
Stankewitz et al. (2009)	Multi-modal rating	Passive	FDR-corrected $p < 0.05$ with an extent threshold of 10	
Treyer et al. (2006)	Odor detection and identification	Passive	Uncorrected $p < 0.001$ at voxel level	
Vedaei et al. (2017)	None	Passive	Uncorrected $p < 0.05$ at cluster level with a Z-threshold of 2.3	
Wang et al. (2017)	None	Active	FWE-corrected $p < 0.001$ with an extent threshold of 6	
Wang et al. (2019)	Odor detection	Passive	Uncorrected $p < 0.001$	
Wiesmann et al. (2006)	None	Passive	Uncorrected $p < 0.001$ at voxel level	

The column 'Inhalation' indicates instruction to participants regarding odor inhalation. Specifically, studies were labeled as 'Active' if participants were clearly instructed to sniff odor stimuli; in contrast, studies were labeled as 'Passive' if participants were not instructed to sniff or were instructed to maintain spontaneous breathing. Abbreviations: FWE, family-wise error; FDR, false discovery rate.

Table S3. Results of the jackknife sensitivity analysis.

	Food		*Non-food*		*Pooled*				*Food > Non-food*
Cluster	1	2	1	2	1	2	3	4	1
Alessandrini et al. (2014)	✓	✓	–	–	✓	✓	✓	✓	✓
Andersson et al. (2014)	✓	✓	–	–	✓	✓	✗	✓	✓
Bengtsson et al. (2001)#1	✓	✓	–	–	✓	✓	✓	✓	✓
Bengtsson et al. (2001)#2	✓	✓	–	–	✓	✓	✓	✓	✓
Eiler et al. (2012)	✓	✓	–	–	✓	✓	✓	✓	✓
Han et al. (2018a)	✓	✓	–	–	✓	✓	✓	✗	✗
Han et al. (2018b)	✓	✓	–	–	✓	✓	✗	✗	✓
Hillert et al. (2007)	✓	✓	–	–	✓	✓	✓	✓	✓
Hoffmann-Hensel et al. (2017)	✓	✓	–	–	✓	✓	✓	✓	✗
Lombion et al. (2009)	✓	✓	–	–	✓	✓	✗	✓	✓
Österbauer et al. (2005)	✓	✓	–	–	✓	✓	✓	✓	✗
Reske et al. (2010)	✓	✓	–	–	✓	✓	✓	✓	✓
Savic et al. (2002)	✓	✓	–	–	✓	✓	✓	✓	✗
Savic et al. (2009)	✓	✓	–	–	✓	✓	✓	✓	✓
Seo et al. (2013)	✓	✓	–	–	✓	✓	✓	✓	✓
Small et al. (1997)	✓	✓	–	–	✓	✓	✓	✓	✗
Tubaldi et al. (2011)	✓	✓	–	–	✓	✓	✓	✓	✗
Zou et al. (2018)	✓	✓	–	–	✓	✓	✓	✓	✓
Ackerley et al. (2020)	–	–	✓	✓	✓	✓	✗	✓	✓
Boyle et al. (2007)	–	–	✓	✓	✓	✓	✓	✓	✓
Eiler et al. (2012)	–	–	✓	✓	✓	✓	✓	✓	✓
Frasnelli et al. (2011)	–	–	✓	✓	✓	✓	✓	✓	✓
Han et al. (2018a)	–	–	✓	✓	✓	✓	✓	✗	✓
Hummel et al. (2013)	–	–	✓	✓	✓	✓	✓	✓	✗
Karunanayaka et al. (2014)	–	–	✓	✓	✓	✓	✓	✓	✓
Karunanayaka et al. (2015)	–	–	✓	✓	✓	✓	✓	✓	✗
Lombion et al. (2009)	–	–	✓	✓	✓	✓	✓	✓	✗
Masaoka et al. (2014)	–	–	✓	✓	✓	✓	✓	✓	✗
Stankewitz et al. (2009)	–	–	✓	✓	✓	✓	✓	✓	✓
Treyer et al. (2006)	–	–	✓	✓	✓	✓	✓	✗	✓
Vedaei et al. (2017)	–	–	✓	✓	✓	✓	✓	✓	✓
Wang et al. (2017)	–	–	✓	✓	✓	✓	✓	✓	✓
Wang et al. (2019)	–	–	✓	✓	✓	✓	✓	✓	✗
Wiesmann et al. (2006)	–	–	✓	✓	✓	✓	✗	✓	✓
Sensitivity	18/18	18/18	16/16	16/16	34/34	34/34	20/34	30/34	23/34

Cluster numbers correspond to the numbers shown in Table 3.

Table S4. ALE meta-analysis using fMRI studies.

Cluster	Volume (mm^3)	Peak locus x	y	z	ALE value	Anatomical label
Food						
1	1,912	22	2	-16	0.0193	Putamen, Lateral globus pallidus, Amygdala, BA34, Medial globus pallidus, BA28
2	1,792	-22	0	-18	0.0201	Amygdala, BA34, Putamen, BA28
Non-food						
1	1,640	26	2	-18	0.0223	Amygdala, BA34, Putamen, Lateral globus pallidus, BA28
2	1,512	-22	-2	-22	0.0250	Amygdala, BA34, BA28
Pooled						
1	3,480	24	2	-18	0.0384	Amygdala, BA34, Putamen, Lateral globus pallidus, Medial globus pallidus, BA28
2	2,952	-22	2	-20	0.0402	Amygdala, BA34, BA28, Putamen
3	968	36	16	2	0.0162	BA13
4	744	-24	32	-10	0.0201	BA47, BA11
*Food > Non-food**						
1	240	18	-2	-16		Medial globus pallidus, Lateral globus pallidus
2	224	-28	6	-14		Putamen
3	8	-34	8	-12		BA13

After all PET studies were excluded, the ALE meta-analyses were performed using the remaining fMRI studies. However, as indicated by the asterisk, a more liberal statistical criterion (i.e., $p < 0.05$ instead of $p < 0.01$) was used in the contrast analysis because of an insufficient statistical power. Abbreviations: ALE, activation likelihood estimation; FWE, family-wise error; FDR, false discovery rate.

REFERENCES

Ackerley, R., Croy, I., Olausson, H., and Badre, G. (2020). Investigating the putative impact of odors purported to have beneficial effects on sleep: neural and perceptual processes. *Chemosensory Perception* 13, 93–105

Alessandrini, M., Micarelli, A., Chiaravalloti, A., Candidi, M., Bruno, E., Di Pietro, B., et al. (2014). Cortico-subcortical metabolic correlates of olfactory processing in healthy resting subjects. *Scientific Reports* 4, 1–6

Andersson, L., Claeson, A.-S., Nyberg, L., Stenberg, B., and Nordin, S. (2014). Brain responses to olfactory and trigeminal exposure in idiopathic environmental illness (IEI) attributed to smells — an fMRI study. *Journal of Psychosomatic Research* 77, 401–408

Bengtsson, S., Berglund, H., Gulyas, B., Cohen, E., and Savic, I. (2001). Brain activation during odor perception in males and females. *Neuroreport* 12, 2027–2033

Boyle, J. A., Heinke, M., Gerber, J., Frasnelli, J., and Hummel, T. (2007). Cerebral activation to intranasal chemosensory trigeminal stimulation. *Chemical Senses* 32, 343–353

Eiler, W. J., Dzemidzic, M., Case, K. R., Considine, R. V., and Kareken, D. A. (2012). Correlation between ventromedial prefrontal cortex activation to food aromas and cue-driven eating: an fMRI study. *Chemosensory Perception* 5, 27–36

Frasnelli, J., Lundström, J. N., Boyle, J. A., Katsarkas, A., and Jones-Gotman, M. (2011). The vomeronasal organ is not involved in the perception of endogenous odors. *Human Brain Mapping* 32, 450–460

Han, J. E., Frasnelli, J., Zeighami, Y., Larcher, K., Boyle, J., McConnell, T., et al. (2018a). Ghrelin enhances food odor conditioning in healthy humans: an fMRI study. *Cell Reports* 25, 2643–2652

Han, P., Mann, S., Raue, C., Warr, J., and Hummel, T. (2018b). Pepper with and without a sting: Brain processing of intranasal trigeminal and olfactory stimuli from the same source. *Brain Research* 1700, 41–46

Hillert, L., Musabasic, V., Berglund, H., Ciumas, C., and Savic, I. (2007). Odor processing in multiple chemical sensitivity. *Human Brain Mapping* 28, 172–182

Hoffmann-Hensel, S. M., Sijben, R., Rodriguez-Raecke, R., and Freiherr, J. (2017). Cognitive load alters neuronal processing of food odors. *Chemical Senses* 42, 723–736

Hummel, T., Olgun, S., Gerber, J., Huchel, U., and Frasnelli, J. (2013). Brain responses to odor mixtures with sub-threshold components. *Frontiers in Psychology* 4, 786

Karunanayaka, P., Eslinger, P. J., Wang, J.-L., Weitekamp, C. W., Molitoris, S., Gates, K. M., et al. (2014). Networks involved in olfaction and their dynamics using independent component analysis and unified structural equation modeling. *Human Brain Mapping* 35, 2055–2072

Karunanayaka, P. R., Wilson, D. A., Vasavada, M., Wang, J., Martinez, B., Tobia, M. J., et al. (2015). Rapidly acquired multisensory association in the olfactory cortex. *Brain and Behavior* 5, e00390

Keller, A. and Vosshall, L. B. (2016). Olfactory perception of chemically diverse molecules. *BMC Neuroscience* 17, 1–17

Lombion, S., Comte, A., Tatu, L., Brand, G., Moulin, T., and Millot, J.-L. (2009). Patterns of cerebral activation during olfactory and trigeminal stimulations. *Human Brain Mapping* 30, 821–828

Masaoka, Y., Harding, I. H., Koiwa, N., Yoshida, M., Harrison, B. J., Lorenzetti, V., et al. (2014). The neural cascade of olfactory processing: a combined fMRI–EEG study. *Respiratory Physiology & Neurobiology* 204, 71–77

Österbauer, R. A., Matthews, P. M., Jenkinson, M., Beckmann, C. F., Hansen, P. C., and Calvert, G. A. (2005). Color of scents: chromatic stimuli modulate odor responses in the human brain. *Journal of Neurophysiology* 93, 3434–3441

Reske, M., Kellermann, T., Shah, N. J., Schneider, F., and Habel, U. (2010). Impact of valence and age on olfactory induced brain activation in healthy women. *Behavioral Neuroscience* 124, 414

Savic, I., Gulyás, B., and Berglund, H. (2002). Odorant differentiated pattern of cerebral activation: comparison of acetone and vanillin. *Human Brain Mapping* 17, 17–27

Savic, I., Hedén-Blomqvist, E., and Berglund, H. (2009). Pheromone signal transduction in humans: What can be learned from olfactory loss. *Human Brain Mapping* 30, 3057–3065

Seo, H.-S., Iannilli, E., Hummel, C., Okazaki, Y., Buschhüter, D., Gerber, J., et al. (2013). A salty-congruent odor enhances saltiness: functional magnetic resonance imaging study. *Human Brain Mapping* 34, 62–76

Small, D. M., Jones-Gotman, M., Zatorre, R. J., Petrides, M., and Evans, A. C. (1997). Flavor processing: more than the sum of its parts. *Neuroreport* 8, 3913–3917

Stankewitz, A., Voit, H., Bingel, U., Peschke, C., and May, A. (2009). A new trigemino-nociceptive stimulation model for event-related fMRI. *Cephalalgia* 30, 475–485

Treyer, V., Koch, H., Briner, H. R., Jones, N. S., Buck, A., and Simmen, D. B. (2006). Male subjects who could not perceive the pheromone 5a-androst-16-en-3-one, produced similar orbitofrontal changes on PET compared with perceptible phenylethyl alcohol (rose). *Rhinology* 44, 279

Tubaldi, F., Turella, L., Pierno, A. C., Grodd, W., Tirindelli, R., and Castiello, U. (2011). Smelling odors, understanding actions. *Social Neuroscience* 6, 31–47

Vedaei, F., Oghabian, M. A., Firouznia, K., Harirchian, M. H., Lotfi, Y., and Fakhri, M. (2017). The human olfactory system: cortical brain mapping using fMRI. *Iranian Journal of Radiology* 14

Wang, J., Sun, X., and Yang, Q. X. (2017). Early aging effect on the function of the human central olfactory system. *Journals of Gerontology Series A: Biomedical Sciences and Medical Sciences* 72, 1007–1014

Wang, K.-W., Chen, C.-Y., Chang, H.-H., Hsu, C.-C., Lan, G.-Y., Hsu, H.-T., et al. (2019). A multivariate empirical mode decomposition–based data-driven approach for extracting task-dependent hemodynamic responses in olfactory-induced fMRI. *IEEE Access* 7, 15375–15388

Wiesmann, M., Kopietz, R., Albrecht, J., Linn, J., Reime, U., Kara, E., et al. (2006). Eye closure in darkness animates olfactory and gustatory cortical areas. *Neuroimage* 32, 293–300

Zou, L., Zhou, H., Zhuang, Y., van Hartevelt, T. J., Lui, S. S., Cheung, E. F., et al. (2018). Neural responses during the anticipation and receipt of olfactory reward and punishment in human. *Neuropsychologia* 111, 172–179
